# Thyroxin Is Useful to Improve Sperm Motility 

**DOI:** 10.22074/ijfs.2016.4911

**Published:** 2016-06-01

**Authors:** Gabriela Ruth Mendeluk, Mónica Rosales

**Affiliations:** 1 Laboratory of Male Fertility, Hospital de Clínicas José de San Martín, Faculty of Pharmacy and Biochemistry, University of Buenos Aires, Buenos Aires, Argentina; 2 Laboratory of Endocrinology, Hospital de Clínicas José de San Martín, Faculty of Pharmacy and Biochemistry, University of Buenos Aires, Buenos Aires, Argentina

**Keywords:** Sperm Motility, Thyroxin, Pentoxifylline, Artificial Insemination

## Abstract

**Background:**

The aim of this study was to evaluate the non-genomic action of thyroxin on sperm kinetic and its probable use to improve sperm recovery after applying an en- richment method like “swim-up” in comparison with the available one, pentoxifylline.

**Materials and Methods:**

This is an experimental study. A total of 50 patients were re- cruited, followed by infertility consultation. Conventional sperm assays were performed according to World Health Organization criteria-2010 (WHO-2010). A Computer Aided Semen Analysis System was employed to assess kinetic parameters and concentrations. Number of the motile sperm recovered after preparation technique was calculated.

**Results:**

Addition of T4 (0.002 µg/ml) to semen samples increased hypermotility at 20 minutes (control: 14.18 ± 5.1% vs. 17.66 ± 8.88%, P<0.03, data expressed as mean ± SD) and remained unchanged after 40 minutes. Significant differences were found in the motile sperm recovered after swim-up (control: 8.93×10^6^ ± 9.52× 06vs. 17.20×10^6^ ± 21.16×10^6^, P<0.03), achieving all of the tested samples a desirable threshold value for artificial insemination outcome, while adding pentoxifylline increased the number of recovered sperm after swim-up in 60% of the studied cases. No synergism between two treatments could be determined.

**Conclusion:**

We propose a new physiological tool to artificially improve insemination. The discussion opens windows to investigate unknown pathways involved in sperm ca- pacitation and gives innovative arguments to better understand infertility mechanisms.

## Introduction

Assisted reproductive technology has been grown by leaps and bounds in the last few years. It is now being increasingly available to infertile couples in both developed and developing countries. In the “Tenth World Report” on assisted reproductive technology, a total of 954743 initiated cycles resulted in an estimated 237809 babies born in 2004. Of all cycles, 60.6% were intracytoplasmic sperm injection (ICSI) (1). The intrauterine insemination (IUI) with husband’s sperm is another assisted reproductive technology, generally believed to be the first choice of treatment rather than more invasive and expensive techniques particularly in the case of cervical infertility, moderate male infertility, dysovulation, mild or moderate endometriosis or unexplained infertility. In the last three indications, ovarian stimulation is necessary. Higher rates of pregnancy obtained by IUI with husband’s sperm and lower risk of multiple pregnancy are related to couple demographic characteristics (age of partners, lifestyle and duration of infertility) and the etiology of infertility (ovarian reserve, uterus, and spermogram). Pregnancy rates were observed ranging from 8 to 20% per cycle according to indications (2). In spite of the huge heterogeneity of patient groups and IUI treatment strategies, it is generally accepted that inseminating motile count after washing should be between 0.8 and 5 million; IUI pregnancy outcome is significantly improved above the latter count, while below the former, a successful outcome is unlikely (3). A recent publication confirmed that total motile spermatozoa inseminated is a predictor of pregnancy rate as well as maternal age and emphasized the impact of the time interval from the end of sperm preparation to IUI on the outcome (4), a variable that probably prevents the deleterious effect of oxidative stress. Adding pentoxifylline, as an inhibitor of cAMP phosphodiesterase enzyme, in semen preparation for IUI improves sperm motility in asthenozoospermic samples due to its capacity to favor increasing intracellular cAMP concentration. Applying this inhibitor has been demonstrated to exert higher and significantly different pregnancy rates in couples classified infertile for male factor (5). 

Asthenozoospermia is probably the main feature in male infertility and has multifactorial origins (6). Although the prevalence of hypotiroidism is higher in females than in males, we wonder about the role of thyronines in sperm motility, considering that they act on nearly every cell in the body increasing the basal metabolic rate. In an experimental design hypothyroid rats showed alterations in the motility of sperm recovered from their epididymis. Transmission electron microscopy technique revealed changes in epididymis epithelial cell’s mitochondria associated with incipient apoptosis, while modifications in proliferative capacity could not be evidenced by argyrophylic proteins of the nucleolar organizer region (7). This technique detects, using silver salts, argyrophylic proteins of the nucleolar organizer region (AgNOR). The number and size of NOR reflect cell activity, proliferation and transformation. Classically thyroid hormones were thought to act in a genomic way. The discovery of a non-genomic effect of these hormones on the cells encouraged us to think in their “*in vitro*” potential actions on sperms. Non-genomic actions of thyroid hormones are independent of nuclear receptors. They have been described at the plasma membrane and cytoplasm, involving changes in solute transport, several kinase activities and effects on specific mRNA translation. Furthermore, they influence the regulation of actin polymerization affecting cytoskeleton dynamics. They also modulate mitochondrial respiration (8). 

The aim of this study was to evaluate the non-genomic action of T4 hormone on sperm kinetic and its probable use to improve sperm recovery after an enrichment method like “swim-up” in comparison with the available one, pentoxifylline. 

## Materials and Methods

### Patients

A total of 50 patients (age: 30-50 years old) consulting for infertility were recruited; males with abnormal thyroid function or using thyroid medication were excluded (clinical history). The present study was conducted according to the guidelines laid down in the “Declaration of Helsinki” and approved by the Institutional Review Board of The Clinical Hospital “José de San Martin”; all the participants received information on the project and gave written informed consent. 

### Design

This is an experimental study and was designed as a prospective, analytical and intervention investigation without randomization. 

### Sperm assays

Conventional sperm assay was performed according to World Health Organization criteria2010 (WHO-2010) (9). A Computer Aided Semen Analysis system (SCA Microptic SL Barcelona, Spain) was employed to assess kinetic parameters and sperm count. The basic components of the system were: a bright field microscope with phase contrast microscopy to visualize the sample (Nikon E200, Japan), a digital camera to capture images (Basler A312 Inc., Vision-Technology, Germany) and a computer with the installed SCA® software. Samples were laid on a thermostatic plate at 37°C. A minimum of 400 sperm cell tracks were captured and 25 digitized images per second were analyzed for each sample. The assays were conducted in accordance with instrument‘s standardization and validation (10), using a Leja chamber 10 (10 µm in depth). A qualified operator validated each analyzed image. 

Data from individual motile spermatozoa, defined by 8 kinematic parameters [curvilinear velocity (VCL), straight-line velocity (VSL), average path velocity (VAP), linearity (LIN), straightness (STR), mean amplitude of lateral head displacement (ALH), wobble (WOB) a measure of oscillation of the actual path about the average path and beat cross frequency (BCF)] were assessed and ratios were then calculated relating the different speeds (LIN=VSL/VCL, SRT=VSL/VAP, WOB=VAP/ VCL). The criteria for detecting hyperactivated spermatozoa was VCL>35 μm/s, ALH>2.5 μm, STR>85%. This CASA criteria for hyperactivation was established by the SCA manufacturer and corresponded to the “transitional activated pattern” (11). 

### Sperm preparation technique: “swim-up”

By the swim up technique, the sperms were selected on their motility and the capability to swim out of the seminal plasma. This is the most prevalently used technique in “*in vitro* fertilization” laboratories and will be preferred if the semen sample is normozoospermic (12). 

The swim-up technique generally produces less reactive oxygen species (ROS) than the other commonly used enrichment technique by using the density-gradient centrifugation, thus generating less sperm DNA damage (13). Briefly after the fluidification of the sample, the semen (well mixed) was divided in fractions of 0.5 ml and transferred into centrifuge tubes. Then, 1.5 ml of culture medium was placed over the semen with extreme attention in each tube, leading to form two phases. The tubes were put in the incubator, inclined at an angle around 45°C and incubated at 37°C for 60 minutes. 

By inclining the tubes at 45°C, we increased the surface between the medium and the semen, improving the capability of the sperms to swim out of the semen and to reach the medium. After that, the tubes were turned back the vertical position and the upper phases of each one were gently aspirated and collected into one tube which was subsequently centrifuged at 600 g for 15 minutes and its volume was adjusted to 0.5 ml. If IUI was performed, 0.3-0.4 ml of spermatozoa suspended in sterile medium would be required. 

Before and after treatment of the seminal fluid, the following parameters were evaluated in line with the WHO Manual 2010: volume (ml), concentration (millions/ml) and motility (progressive motility) (9). The concentration of the progressive spermatozoa (PS) is calculated by multiplying the percentage of the progressive sperms (PS%) and the concentration of the sperms (S) in the final preparation. Total number of the PS (TPS) is calculated by multiplying (PS) and the volume (V) in the final preparation. 

$$[\mathrm{PS}]=\%\mathrm{PS}\times {\left[S\right]}_{\mathrm{final}}$$

$$\mathrm{TPS}=[\mathrm{PS}]\times V_{\mathrm{final}}$$

The total number of the progressive sperms in the preparation, before IUI, might be defined as a threshold value in predicting IUI outcome. The accepted cut-off value is five millions (3).

Capacitating medium was composed of HAM F-10 1x (+25 mM HEPES and L-glutamine, GIBCO-Life Technology, USA) supplemented with penicillin-streptomycin (10000 U/ml penicillin, 10 ml µg/ml streptomycin, both obtained from GIBCO-Life Technology, USA) and serum substitute supplement (Irvine Scientific, California, USA). 

### Improving sperm enrichment technique

Following the experimental design proposed by Nassar et al. (14), we compared the effect of pentoxifylline and T4 (Levotiroxina Montpellier 200 µg/ml) on sperm recovery after swim-up. 

The recovery rate was calculated as follows: 

$$\%\mathrm{Recovery rate}=\frac{\%{\mathrm{PS}}_{\mathrm{final}}\times V_{\mathrm{final}}\times {\left[S\right]}_{\mathrm{final}}}{\%{\mathrm{PS}}_{\mathrm{initial}}\times V_{\mathrm{initial}}\times {\left[S\right]}_{\mathrm{initial}}}\times 100$$

In this equation, initial and final means sperm parameters before and after the “swim-up”, respectively.

### Statistical analysis

Evaluation results after supplementation was based on Student t test (MedCal). P values of less than 0.05 were considered statistically significant. 

## Results

### Choosing an optimal dose of T4

We used a semen sample with exceeding the normal cut-off value established by WHO, showing sperm concentration of >179.6×10^6^ sperm/ml, sperm progressive motility of 63.9%, sperm morphology of 23%, sperm vi- tality of 85% and sperm volume of 4.8 ml. The sample was diluted 1/10 to achieve an optimal concentration for kinetic studies. Kinetic pa- rameters were evaluated after the adding T4 (Levotiroxina Montpellier, 200 µg/ml). Hor- mone dilutions ranging from particular serum concentration (0.1 µg/ml) to the expected seminal plasma concentration (0.001 µg/ml) were tested [final concentration of 0.2 µg/ml (Dil.: 1), 0.002 µg/ml (Dil.: 2), 0.00002 µg/ ml (Dil.: 3), 0.00000002 µg/ml (Dil.: 4). The undiluted hormone showed cytotoxicity for the sperm, represented by complete immotil- ity and necrozoospermia (Vital test). In our system, Ga was defined with VCL>35 µm/s. Lineality>50% and Straightness>80%. Hor- mone dilution of 2 (Dil.: 2) was determined to improve Ga sperm motility, suggesting eli- gibility of this T4 concentration (basal n=307: 35.2%; Dil. 1. n=335: 35.2%; Dil. 2. n=357: 44.5%; Dil 3. n=427: 34.9%; Dil 4. n=423: 34%). 

### Non genomic effect of T4 on sperm kinetic

Semen samples were fractioned in two aliquots of 0.5ml (n: 17). Dilution 2 was added to the first one, applied as the second control of the assay. Sperm kinetic was then evaluated after 20 and 40 minutes. We only detected parameter differences between untreated aliquot and hyperactivity, leading to significantly increase after 20 minutes (control: 14.18 ± 5.1% vs. 17.66 ± 8.88 %, P<0. 03, data expressed as mean ± SD) and remained unchanged after 40 minutes. 

### Appropriate time to add the hormone

The previous results encouraged us to employ the hormone in the sperm preparation for IUI. In ten semen samples dilution 2 was added twenty minutes before performing the swim-up and at the time of swim-up. No significant difference was detected between treatments, while evaluating the recovery rate (%): 33.07 ± 22.4 vs. 36.63 ± 24 (data expressed as mean ± SD). In order to facilitate the procedure and avoid time delay and ROS production, the second option was employed in future experiments. 

### Testing the hormone

Semen samples were fractioned in two aliquots of 0.45 ml each (n: 17). Hormone dilution was prepared at that moment preventing oxygen and light effect [0.02 µg/ ml in phosphate-buffered saline (PBS) with pH=7.2-7.4].Working in sterility, to avoid contamination, 50 µl of the freshly prepared hormone was added to the first aliquot. No hormone was added to the second aliquot, as the control of test group. Significant differences were found in the number of motile sperms recovered after procedure in the studied groups (control: 2.16×10^6^± 2.55×10^6^vs. 3.00×10^6^ ± 2.6×10^6^, P<0.03, data expressed as mean ± SD).

### Improving the chances for intrauterine insemination outcome

Total number of the progressive sperms in the preparation before the IUI could be defined as a threshold value in predicting outcome in IUI. The accepted cut-off value is five millions (3). 

Table 1 shows projection of the results evaluated in the former experiment, in case of using complete sample (i.e. whole volume of the ejaculate, as a procedure currently performed in andrology laboratories prior to IUI). Significant differences were found in the number of motile sperm recovered after this procedure in the studied groups (control: 8.93×10^6^± 9.52×10^6^vs. 17.20×10^6^ ± 21.16×10^6^, P<0. 03, data expressed as mean ± SD).

From 17 tested semen samples, 14 increased the number of recovered sperms, reaching the desired threshold value for IUI outcome. Notably, only in three cases a negative difference was observed, due to outstanding semen smears, whereby no needs of improvement methods are required. 

**Table 1:** Differences in the total motile sperm recovered with/without T4 hormone in each patient, by using the whole ejaculate


Total motile sperm without T4 (×10^6^)	Total motile sperm with T4 (×10^6^)	Outcome differences between procedures (×10^6^)

3.61	10.71	7.09
3.61	4.46	0.86
3.04	6.27	3.22
1.91	4.84	2.93
7.12	8.93	1.81
19.82	15.76	-4.06
36.64	86.17	49.53
15.56	17.19	1.63
71.13	50.95	-20.18
3.75	8.09	4.34
4.40	13.22	8.82
5.83	13.13	7.29
3.73	16.61	12.89
84.11	65.34	-18.76
16.92	41.37	24.45
4.43	5.60	1.17


### Comparing the effect of the proposed T4 method with pentoxifylline

**Fig.1:**
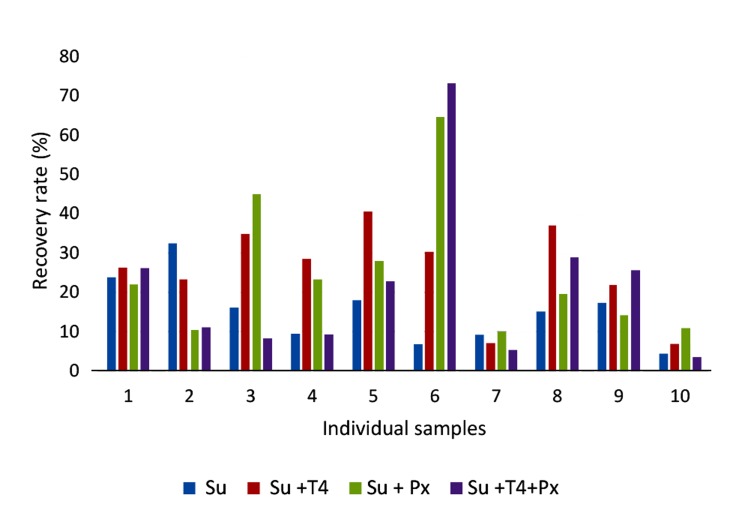
Comparison of swim-up recovery rate (%) among procedures. Data illustrates swimm-up recovery rate (%) in ten individual samples. Basal swim-up (Su), swim-up with T4 (Su+T4) P<0.01, swim-up with pentoxifylline (Su+Px) and swim-up with T4 and pentoxifylline (Su+T4+Px).

Semen samples were fractioned in four aliquots (n: 10). The first aliquot was served as the control group. T4, with final concentration of 0.002 µg/ml, was added to the second aliquot. pentoxifylline (fi- nal concentration of 1 mg/ml) was added to the third aliquot and the synergism between these two treat- ments was evaluated in the fourth aliquot. Swim-up method was then performed and the recovery rate was calculated. Findings showed that T4 treatment was statistically superior to the control (control: 15.31 ± 8.46% vs. 25.71 ± 11.46%, P<0.01, data expressed as mean ± SD). No significant difference was observed by comparing the control with treated pentoxifylline group (control: 15.31 ± 8.46% vs. 24.85 ± 17.56%, data expressed as mean ± SD). In addition, we did not find any significant difference between control group and the treated samples with both T4 and pentoxifylline (control: 15.31 ± 8.46% vs. 21.47 ± 20.51%). Out of ten, the recovery rate was improved in eight samples by using T4 treat- ment (80%), while this improvement was observed only in six samples (60%) by treating with pentoxi- fylline (Fig .1). No synergism was detected between these types of treatment in our experiments.

## Discussion

In this study, treating semen samples with T4 generated a significant increase in the percentage of hyperactive sperm. The effect of this hormone appears to happen rapidly, since there was no change between data on the objective motility after 20 and 40 minutes. The immediate action of this hormone oriented us to propose a non-genomic action of T4, as a novel concept which might change the current Mendeluk and Rosales investigation approaches in fertility. More beyond that and aligned with previous studies from our group, demonstrating the positive effect of pentoxifylline induction on the hyperactivity and quantity of progressive motile sperms after swim-up (15), at basal time, encouraged us toward investigating the potential effect of T4, as a novel agent, on optimization of the sperm preparation for IUI in the fertilization laboratories. In this experiment, we demonstrated that our hypothesis was correct, evidenced by the improvement of sperm count after swim-up. Thus, a patent was then presented in our country for this finding on April 13 ^th^2013. titled: “Método *in vitro* para la capacitación de espermatozoides. Instituto Nacional de la Propiedad Industrial. Administración Nacional de Patentes. INPI Exp: 20130101645. Inventores: Gabriela R. Mendeluk; Mónica Rosales; Mercedes N. Pugliese; Patricia H. Chenlo”. 

In this study, we did not detect any synergism between pentoxifylline and T4. Similar to the previous study, treatment with pentoxifylline increased the number of recovered sperms after swim-up in 60% of the cases, from a mathematical point of view. Focusing on a program of assisted reproduction, at least 5 million progressive motile sperms are needed for IUI outcome. From a clinical point of view, all of the studied samples overcame this barrier by employing T4. Interestingly, overcoming the cut-off value is the only negative result obtained by comparing swim-up outcome treated with or without the hormone, suggesting that there is an upper motility limit, above which adding the hormone has no effect. Despite the proposed method is reasonable for subfertil sperms, it could not be applicable for severely impaired semen samples, as an alternative beyond ICSI. This is in accordance with findings obtained from the target group which has been benefited by this treatment. 

Thyroid hormones can exert their actions at different cellular levels: within the cell nucleus, at the plasma membrane, in cytoplasm, and at the mitochondrion. Regarding the fact that sperm nucleus is compacted, the first option was discarded in this study. Actions of thyroid hormone that are not initiated by liganding hormone to intranuclear thyroid receptors are termed nongenomic. Nongenomic actions are independent from protein synthesis, thus inducing short time responses. They may trigger plasma membrane receptors like αVβ3 integrin, activating signal transduction via kinase pathways or modifying ion fluxes and membrane potential. They can also act by specific binding sites located in the cytosol. This mechanism is related to cytoskeleton dynamic, since they promote actin polymerization (16). 

In 1996, Fusi et al. (17) reported that expression of specific integrin chains were altered with the functional state of spermatozoa. The cells displaying beta 3 integrin were correlated with a proportion of spermatozoa which had undergone an acrosome reaction, following calcium ionophore exposure. Boissonnas et al. (18) demonstrated the presence of αVβ3 integrin by performing Western blot and immunofluorescence techniques on the sperm membrane. Using the previous statements, we can infer that there is a non-genomic receptor for T4 hormone, at least in sperm membrane. The molecular mechanisms of the effect of this hormone on sperm kinetic are still difficult to evaluate. Our hypothesis, based on what have yet been determined about the non-genomic effect of T4 on ion channels, is that it increases Ca ^2+^intake (19) which in the HCO3 ^−^environment stimulates atypical sperm adenylyl cyclase. Finally, generation of cAMP could increase protein kinase A (PKA) activity at the molecular level leading to vigorous flagellar movement, called “hyperactivation” (20,21). Pentoxifylline, as a phosphodiesterase inhibitor, potentiate cAMP lifespan by preventing degradation of this molecule. Indeed, it acts in the last part of this cascade, while T4 is involved on the upstream having a major role in the phenomenon. More beyond that, and in this context, thyroid hormones also act upstream of phospholipase C (PLC), forming diacylglycerol (DAG) and inositol 3.4.5-phosphate (IP3), while the latter molecule has a capacity to release Ca ^2+^from intracellular stores feeding back the process. Consistent to this, we could explain the nonsynergism and probable complementation between T4 and pentoxifylline, as well as inferior recovery rates obtained by using the latter agent. 

In females, hypothyroidism is mainly associated with oligomenorrhea. Several adverse reproduction outcomes are related to overt hypothyroidism, such as increased incidence of spontaneous abortions, placental abruption, fetal distress in labor, premature delivery and/or low birth weight, as well as gestation-induced hypertension (22). Thyroid dysfunction has also been linked to reduced fertility. Hence, it should be further investigated the fact whether adding T4, as with *in vitro*, could be effectively supplied in vivo. It is proposed that by lack of this agent administration, the sperm would have less chance to acquire hyperactivated status, as a physiological and mechanical requirement to improve fertilization outcome. In our opinion, the hormonal thyroid status, either in male or female, should be considered while evaluating the predictive factors for pregnancy in case of IUI administration (23). 

It has been proposed that IUI is less expensive, less invasive, and comparably effective for selected patients, as a first-line treatment for couples (24). We are currently suggesting a new physiological tool to improve this technique which requires further evaluation to validate its potentially effective application in reproduction. The discussion opens our minds to think in unknown pathways involved in sperm capacitation and gives innovative arguments to better understand infertility mechanisms. 

## Conclusion

Our report is the first, to our knowledge to envisage a non-genomic action of thyroxine on sperm with direct impact on clinical. 

## References

[1] Merviel P, Cabry R, Lourdel E, Barbier F, Scheffler F, Mansouri N (2014). [Intrauterine insemination]. Rev Prat.

[2] Sullivan EA, Zegers-Hochschild F, Mansour R, Ishihara O, de Mouzon J, Nygren KG (2013). International Committee for Monitoring Assisted Reproductive Technologies (ICMART) world report: assisted reproductive technology 2004. Hum Reprod.

[3] Ombelet W, Dhont N, Thijssen A, Bosmans E, Kruger T (2014). Semen quality and prediction of IUI success in male subfertility: a systematic review. Reprod Biomed Online.

[4] Fauque P, Lehert P, Lamotte M, Bettahar-Lebugle K, Bailly A, Diligent C (2014). Clinical success of intrauterine insemination cycles is affected by the sperm preparation time. Fertil Steril.

[5] Negri P, Grechi E, Tomasi A, Fabbri E, Capuzzo A (1996). Effectiveness of pentoxifylline in semen preparation for intrauterine insemination. Hum Reprod.

[6] Curi SM, Ariagno JI, Chenlo PH, Mendeluk GR, Pugliese MN, Sardi Segovia LM (2003). Asthenozoospermia: analysis of a large population. Arch Androl.

[7] Palaoro LA, Rocher AE, Canessa OE, Peressini S, Rosales M, Del Río AG (2013). Epididymal mitochondrial status of hypothyroid rats examined by transmission electron microscopy. Biotech Histochem.

[8] Davis PJ, Davis FB (1996). Nongenomic actions of thyroid hormone. Thyroid.

[9] World Health Organization (2010). WHO laboratory manual for the examination and processing of human semen.

[10] Chenlo P, Ariagno JI, Pugliese MN, Repetto HE, Sardi Segovia L, Mendeluk GR (2013). Study of human semen: implementation of an objective method. Acta Bioquím Clín Latinoam.

[11] Suarez SS (1996). Hyperactivated motility in sperm. J Androl.

[12] Björndahl L, Mohammadieh M, Pourian M, Söderlund I, Kvist U (2005). Contamination by seminal plasma factors during sperm selection. J Androl.

[13] Zini A, San Gabriel M, Baazeem A (2009). Antioxidants and sperm DNA damage: a clinical perspective. J Assist Reprod Genet.

[14] Nassar A, Mahony M, Morshedi M, Lin MH, Srisombut C, Oehninger S (1999). Modulation of sperm tail protein tyrosine phosphorylation by pentoxifylline and its correlation with hyperactivated motility. Fertil Steril.

[15] Mendeluk GR, Chenlo PH, Sardi-Segovia M, Curi S, Ariagno J, Repetto H (2010). Usefulness of pentoxifylline to improve semen quality. Fertil Steril.

[16] Cheng SY, Leonard JL, Davis PJ (2010). Molecular aspects of thyroid hormone actions. Endocr Rev.

[17] Fusi FM, Tamburini C, Mangili F, Montesano M, Ferrari A, Bronson RA (1996). The expression of alpha v, alpha 5, beta 1, and beta 3 integrin chains on ejaculated human spermatozoa varies with their functional state. Mol Hum Reprod.

[18] Boissonnas CC, Montjean D, Lesaffre C, Auer J, Vaiman D, Wolf JP (2010). Role of sperm alphavbeta3 integrin in mouse fertilization. Dev Dyn.

[19] Zamoner A, Pessoa-Pureur R, Silva FR (2011). Membrane-initiated actions of thyroid hormones on the male reproductive system. Life Sci.

[20] Visconti PE (2009). Understanding the molecular basis of sperm capacitation through kinase design. Proc Natl Acad Sci USA.

[21] Navarrete FA, García-Vázquez FA, Alvau A, Escoffier J, Krapf D, Sánchez-Cárdenas C (2015). Biphasic role of calcium in mouse sperm capacitation signaling pathways. J Cell Physiol.

[22] Krassas GE, Poppe K, Glinoer D (2010). Thyroid function and human reproductive health. Endocr Rev.

[23] Merviel P, Heraud MH, Grenier N, Lourdel E, Sanguinet P, Copin H (2010). Predictive factors for pregnancy after intrauterine insemination (IUI): an analysis of 1038 cycles and a review of the literature. Fertil Steril.

[24] Abdelkader AM, Yeh J (2009). The potential use of intrauterine insemination as a basic option for infertility: a review for technology-limited medical settings. Obstet Gynecol Int.

